# Characteristics of Combined Movement Behavior Interventions in Children and Adolescents: A Scoping Review

**DOI:** 10.1111/obr.13943

**Published:** 2025-06-04

**Authors:** Ciaran M. C. Maloney, Andrew J. Atkin, Lee C. Beaumont, Emily Budzynski‐Seymour, Victoria E. Warburton

**Affiliations:** ^1^ School of Education and Lifelong Learning University of East Anglia Norwich UK; ^2^ School of Health Sciences University of East Anglia Norwich UK; ^3^ Norwich Medical School University of East Anglia Norwich UK

**Keywords:** intervention, physical activity, sedentary behavior, sleep

## Abstract

Evidence suggests that targeting physical activity, sedentary behavior, and sleep in combination can benefit health and academic outcomes in young people. This scoping review aimed to describe the extent, range, and nature of combined movement behavior interventions and examine recruitment and effectiveness patterns in equity‐denied populations. The following electronic databases were searched: Web of Science, CINAHL, MEDLINE, and PsycINFO. Gray literature was identified through ProQuest Dissertations and Theses Global, Google Scholar, and the British Library EThOS. Included studies were randomized or quasi‐experimental interventions that modified two or more movement behaviors with the goal of affecting health‐, behavioral‐, or academic‐related outcomes in children or adolescents. Peer‐reviewed publications from scientific databases, master's level dissertations, and doctoral theses from gray literature searches in the English language were included. The behavior change technique taxonomy and PROGRESS‐Plus framework were used to map intervention characteristics. Thirty studies met the inclusion criteria. Most studies were individual‐level randomized controlled trials (40%), conducted in Europe (43%), and delivered in a school setting (77%). Physical activity and sedentary behavior were the predominant behaviors that were modified (83%). The most commonly used behavior change techniques included information about health consequences (67%) and social support [unspecified] (70%). All included studies focused on health‐related outcome measures. Ten studies (33%) examined differential effects by PROGRESS‐Plus subgroups. Future research should explore the value of movement behavior interventions across the breadth of non‐health‐related outcomes and include a stronger focus on differential effectiveness across population subgroups.

## 1 Introduction

A wide‐ranging evidence base describes the individual health impacts of physical activity [1], sedentary behavior [2], and sleep [3] in young people. In recent years, however, there has been a shift in epidemiological and behavioral science research towards understanding the combined influence of these behaviors on health, sometimes referred to as the 24‐h movement behavior paradigm [4]. Within a fixed 24‐h daily schedule, any time allocated to physical activity, sedentary behavior, and sleep necessitates simultaneous duration adjustments in the other behaviors [5, 6, 7]. This understanding has led to the development of 24‐h movement behavior guidelines for young people in several countries, including Canada [8] and Australia [9], though the suitability of evidence underlying such guidelines has been questioned [10].

Young people who engage in high levels of physical activity, obtain sufficient sleep, and have low sedentary behavior tend to attain better academic grades [11] and experience several health benefits [12, 13], including lower body mass index [14], improved mental health [15], and a positive trajectory towards better adult health [16]. However, many young people do not meet movement behavior guidelines [17, 18, 19], particularly those from equity‐denied populations, including low socioeconomic groups [20]. For example, children with less educated parents or lower family income tend to accumulate less physical activity [21], spend more time sedentary [22], and are more likely to get insufficient sleep [23] compared to those from more affluent families. Childhood and adolescence are critical periods to reduce inequalities in movement behavior patterns as behaviors established during these years tend to persist into adulthood [24].

The shift towards studying movement behaviors in combination has increased interest in the potential health benefits of multiple health behavior change interventions [25]. Previous reviews of multiple health behavior change interventions in young people have principally focused on effectiveness [26, 27], with limited description of intervention characteristics. For example, a recent review of school‐based, multiple lifestyle behavior interventions showed that such programs can elicit changes in physical activity, screen time, and diet in the short term [26], but this review did not examine whether effects differed between participant subgroups. Previous research has shown that under certain circumstances, public health interventions may inadvertently widen health disparities by primarily benefiting advantaged, low‐risk populations rather than equity‐denied, higher‐risk groups [28, 29, 30]. “Highly agentic” interventions, for example, which require participants to use their personal resources or “agency” have been shown to reinforce or exacerbate inequalities in dietary behaviors [29, 30]. At present, there is limited knowledge about the differential effectiveness of combined movement behavior interventions [31].

A comprehensive overview of the intervention landscape serves to highlight areas of limited evidence and aid the design of future interventions. Therefore, this scoping review aimed to describe: (1) the extent, range, and nature of combined movement behavior interventions in young people; and (2) the recruitment and effectiveness patterns in equity‐denied population groups.

## 2 Method

This scoping review was conducted in accordance with guidance from the JBI [32] and reported using the Preferred Reporting Items for Systematic Review and Meta‐Analysis extension for Scoping Reviews checklist [33] (Supplementary Material 1). The review protocol was registered on Open Science Framework (doi.org/10.17605/OSF.IO/86EXB).

### 2.1 Eligibility Criteria

Randomized controlled trials and quasi‐experimental studies that modified two or more movement behaviors with the goal of affecting health‐, behavioral‐, or academic‐related outcomes were included. Relevant interventions targeted movement behaviors as the intervention modality, manipulating them directly or modifying their determinants, with the goal of eliciting change(s) in the specified outcome(s). This encompassed interventions that additionally modified other health‐related behaviors, such as diet, smoking, and alcohol consumption. Studies were eligible if they focused on children (aged 5–11 years) and/or adolescents (aged 12–24 years) at baseline. Studies that included participants outside the 5–24‐year age range were included if the results were reported separately for eligible age groups or if the mean age was within this range. No limits were set on publication date, intervention duration, setting, or mode of delivery. Master's dissertations, doctoral theses, and articles from peer‐reviewed journals were included. Articles published in languages other than English were excluded. Commentaries, conference papers, previous reviews, and undergraduate dissertations were also excluded.

### 2.2 Search Strategy and Information Sources

An initial search of Web of Science and MEDLINE Complete (EBSCO) was performed to identify an indicative subset of articles on the topic. Article titles, abstracts, and author‐specified keywords were used to develop the search strategy. The search strategy was developed with input from an academic librarian and adapted for each database (Supplementary Material 2). Searches were initially conducted in June 2022 and repeated in October 2023. Databases searched were Web of Science, PsycInfo (EBSCO), MEDLINE Complete (EBSCO), and Cumulative Index to Nursing and Allied Health Literature (CINAHL; EBSCO). As recommended [34, 35], gray literature was identified through searches of ProQuest Dissertations and Theses Global, Google Scholar, and the British Library EThOS, respectively. The reference lists of relevant review articles [26, 27, 36, 37, 38, 39, 40] and included records were screened to identify any additional articles not identified through database searches.

### 2.3 Selection of Sources of Evidence

Search results were collated and uploaded into EndNote X9 (Clarivate, Pennsylvania, United States), where duplicates were removed using the “duplicate” function and manually by the lead author. Two reviewers (CM and EB‐S) independently screened the title and abstract of 100 search records in Rayyan [41] to ensure inclusion and exclusion criteria were unambiguous and applied consistently. Following the pilot test, all records were exported to Covidence Systematic Review Software [42] for title and abstract screening, conducted in duplicate by two reviewers (CM and EB‐S). Full‐text manuscripts were retrieved based on title and abstract screening (CM and EB‐S; proportionate agreement = 92%). As above, full‐text articles were independently screened for inclusion by two reviewers (CM and EB‐S; proportionate agreement = 75%). For both title/abstract and full‐text screening, conflicts that could not be resolved through discussion between primary reviewers were resolved through discussion with an additional reviewer (AA).

### 2.4 Data Items and Data Charting Process

A data charting template was drafted by the lead author with input from the review team. To ensure consistency, the template was piloted in five studies by two members of the review team (CM and EB‐S). The lead author (CM) charted data from all studies and 20% were randomly selected and extracted in duplicate by a second team member (EB‐S). Data charted from each study was in line with guidance from the template for intervention description and replication (TIDieR) checklist [43]. Participant characteristics were extracted according to dimensions outlined in the PROGRESS‐Plus framework [44]. The framework denotes place of residence, race, occupation, gender, religion, education, socioeconomic status, social capital, and any other vulnerable characteristic, enabling us to ascertain whether studies sought to recruit and assess intervention effectiveness in equity‐denied populations. The following information was charted: study and participant characteristics (e.g., citation details, sample size, mean age, biological sex, and population described by PROGRESS‐Plus factors), intervention characteristics (e.g., setting and provider, delivery and duration, theoretical framework, targeted behaviors, behavioral change techniques used [details below], process evaluation, outcomes) and reporting effectiveness by PROGRESS‐Plus factors.

The Behaviour Change Technique Taxonomy v1 [45] was used to record which behavioral change techniques were used in included studies. The framework comprises 93 unique behavioral change techniques grouped into 16 hierarchical clusters. Twenty percent of the included studies were independently mapped onto the Behaviour Change Technique Taxonomy v1 by two reviewers (CM and AA) and any disparities were discussed. Subsequently, the remaining studies were coded by the lead author (CM). A behavioral change technique was coded only if there was clear evidence of inclusion. Where available, study protocols were reviewed and used to inform the recording of behavioral change techniques.

### 2.5 Synthesis of Results

Study was the unit of analysis. Synthesis was narrative and in the form of summary tables. Consistent with our study aims, the synthesis described the characteristics of included studies (e.g., year of publication, study design, sample), intervention characteristics (e.g., setting, targeted behaviors, behavioral change techniques, outcomes, evaluations) and differential effectiveness by PROGRESS‐Plus subgroups.

## 3 Results

**FIGURE 1:**
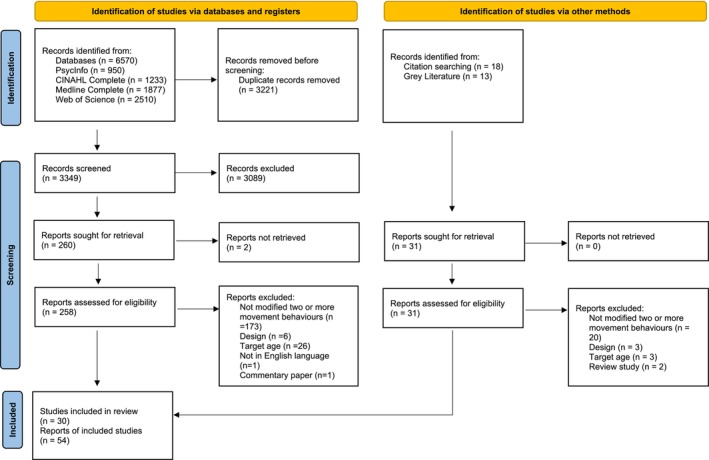
Flow diagram of article selection process.

As depicted in Figure 1, 6570 records were identified from the database search. After removal of duplicates, the titles/abstracts of 3349 records were screened, of which 258 were retrieved for full‐text appraisal. Citation and gray literature searching yielded 31 records. In total, 30 studies, described across 54 publications, met the inclusion criteria. Of the 30 included studies, 27 were identified through database searches and three were identified through other search methods.

### 3.1 Study and Participant Characteristics

Characteristics of included studies are presented in Table 1. Of the 30 included studies, 13 (43%) were conducted in Europe, eight (27%) in North America, four (13%) in Australia and Oceania, three (10%) in Asia, and two (7%) in South America. Most studies were individual‐level randomized controlled trials (*n* = 12, 40%), 11 (37%) were cluster randomized controlled trials, and seven (23%) used a quasi‐experimental design. Year of publication ranged from 2006 to 2023, but over half (*n* = 17, 57%) were published in the last 5 years and over 80% in the last 10 years (*n* = 25, 83%). Sample sizes ranged from 48 to 9280; median (IQR) was 361 (194–825.5).

**TABLE 1:** Study and participant characteristics (presented separately for studies in children and adolescents).

First author (publication date), country	Design (number of arms)	Control conditions	Sample size recruited (analyzed)	Age; mean (SD)	Gender % (male/female)	Population described by PROGRESS‐Plus factors
Children						
Ahmad [46] (2018), Malaysia	RCT (two‐arm)	Wait‐list control group	134 (134)	9.6 (1.2)	E:40/60 C:43/57	Gender, education, SEP, age
Bergh [47] (2012), Norway	CRCT (two‐arm)	Regular school curriculum	2165 (1418)	E:11.02 (0.27) C:11.02 (0.26)	E:50/50 C:52/48	Gender, education, SEP, age
Bernal [48] (2021), France	RCT (two‐arm)	No intervention	181 (181)	E:8.66 (1.36) C:8.87 (1.40)	E:60/40 C:40/60	Gender, SEP, age
Carson [49] (2013), Australia	CRCT (four‐arm: 3E, 1C)	Regular school activities	599 (293)	8 (1.3)	44/56	Gender, education, SEP, age
De Lepeleere [50] (2017), Belgium	QE (two‐arm)	No intervention	207 (207)	E:9.2 (1.5) C:9.6 (1.6)	E:55/45 C:48/52	Gender, SEP, age
Faghy [51] (2021), United Kingdom	QE (one‐arm)	No control group	147 (147)	8.9 (1.3)	50/50	Gender, age
Harrison [52] (2006), Ireland	QE (two‐arm)	Health Education Curriculum	312 (312)	E:10.2 (1.2) C:10.3 (0.8)	E:56/44 C:58/42	Gender, SEP, age
Jago [53] (2013), United Kingdom	RCT (two‐arm)	No intervention	48 (48)	E:6.6 (1.3) C:8 (1.89)	E:38/62 C:31/69	Race, gender, education, SEP, age
Kipping [54] (2014), UK	CRCT (two‐arm)	Regular school activities	2221 (1825–2121)	9.5 (0.3)	E:51/49 C:48/52	Gender, SEP, age
Moore [55] (2019), United States	RCT (three‐arm: 2E, 1C)	Received brief education on PA and healthy eating	360 (360)	11.6 (0.6)	42/58	Race, education, SEP, age
Puder [56] (2011), Switzerland	CRCT (two‐arm)	Regular school activities	652 (652)	5.2 (0.6)	50/50	Gender, age
Robinson [57] (2021), United States	RCT (two‐arm)	Health education intervention	241 (241)	9.5 (1.4)	44/56	Race, gender, SEP, age
Salmon [58] (2008), Australia	RCT (four‐arm: 3E, 1C)	Regular school activities	311 (306)	10.8 (0.5)	49/51	Gender, SEP, age
Siegrist [59] (2018), Germany	CRCT (two‐arm)	Regular activities	558 (434)	11.1 (0.6)	57/43	Race, gender, age
Tapia‐Serrano [60] (2022), Spain	QE (two‐arm)	Ten 1‐h sessions per week without health intervention	121 (121)	9.01 (0.09)	53/47	Gender, SEP, age
Vilardell‐Davila [61] (2023), Mexico	QE (two‐arm)	Received general recommendations to improve PA and healthy eating	368 (368)	8.0 (SD not reported)	53/47	Gender, education, SEP, age
Adolescents						
Ahmed [62] (2022), Bangladesh	CRCT (two‐arm)	No intervention	320 (320)	E:14.42 (1.15) C:14.18 (0.89)	E:66/34 C:52/48	Gender, education, SEP, age
Aittasalo [63] (2019), Finland	RCT (two‐arm)	Adjusted their health education lessons on PA to the same time‐period as intervention	1550 (1476)	13.9 (0.5)	E:52/48 C:53/47	Place of residence, gender, age
Bandeira [64] (2020), Brazil	CRCT (two‐arm)	Regular school activities	1085 (1085)	Not reported	E:52/48 C:51/49	Gender, SEP, age
Bonnesen [65] (2021), Denmark	CRCT (two‐arm)	No intervention	4577 (4577)	16.2 (1.1)	37/63	Gender, age
Champion [66] (2023), Australia	CRCT (two‐arm)	Regular health education	9280 (6640)	12.7 (0.50)	E:52/47^a^ C:48/50^a^	Gender, education, SEP, age
Choi [67] (2018), Korea	QE (two‐arm)	Given brief health promotion instructions	63 (63)	21.2 (2.63)	37/63	Gender, SEP, age
Filion [68] (2015), United States	RCT (two‐arm)	Comparison smoking cessation group received 6‐week intervention	164 (116)	21.8 (2.1)	53/47	Race, gender, age
Knebel [69] (2020), Brazil	CRCT (two‐arm)	Regular school activities	1427 (597)	13 (1)	E:49/51 C:46/54	Gender, age
Laska [70] (2016), United States	RCT (two‐arm)	Received basic health information	441 (441)	22.8 (not reported)	32/68	Race, gender, SEP
Mauriello [71] (2010), United States	RCT (two‐arm)	No intervention	1800 (1800)	15.97 (not reported)	49/51	Race, gender, age
Sevil‐Serrano [72] (2019), Spain	QE (two‐arm)	No intervention	225 (210)	E:13.05 (0.59) C:13.07 (0.63)	E:48/52 C:47/53	Gender, SEP, age
Smith [73] (2014), Australia	CRCT (two‐arm)	Regular school activities	361 (361)	12.7 (0.5)	100/0	Race, gender, SEP, age
Werch [74] (2011), United States	RCT (two‐arm)	Received a standard care control booklet	479 (479)	17 (0.82)	38/62	Race, gender, SEP, age
Young [75] (2022), United States	RCT (two‐arm)	Wait‐list control group	51 (37)	E:18.5 (2.5) C:17.4 (2.3)	0/100	Race, gender, SEP, age

Abbreviations: C, control; CRCT, cluster randomized controlled trial; E, experimental; PA, physical activity; QE, quasi‐experimental; RCT, randomized controlled trial; SEP, socioeconomic position.

^a^ This study included other gender categories, which account for the remaining percentages.

Sixteen studies (53%) targeted children (age range 5–11 years), 11 (37%) targeted adolescents (age range 12–24 years), and three studies (10%) targeted both age groups. All studies described participants' age and sex. Twenty‐two studies (73%) reported an indicator of participants' socioeconomic position (household/family income) or school‐/area‐level socioeconomic position (index of multiple deprivation, location of schools and residential postcodes, qualification of free school meals, and family affluence scale). Eleven studies (37%) reported participant ethnicity, eight studies (27%) reported parents' level of education, and one study (3%) reported place of residence (type of residency building).

### 3.2 Intervention Characteristics

#### 3.2.1 Setting, Delivery, and Duration

Intervention characteristics are described in Table 2. Twenty‐three (77%) interventions were delivered in a school setting and two (7%) at a university. Two studies (7%) implemented interventions via a hybrid approach (i.e., in‐person and online/telephone/social media), and single studies were implemented online, in a community center, and via text messaging. Most interventions were delivered face‐to‐face (*n* = 21, 70%), with the remainder being delivered online in the home (*n* = 1, 3%), online in a school setting (n = 1, 3%), hybrid (*n* = 6, 20%), or via text messaging (*n* = 1, 3%). Teachers (*n* = 17, 57%) and researchers (*n* = 8, 27%) predominantly delivered interventions, while two studies (7%) used interventionists (leisure center personnel/individuals trained in psychology, nursing, or social work). Single studies used nurses and therapists, nutritionists and physical educators, and coaches. Parents were also involved in 18 studies (60%) through receiving intervention materials and/or interacting in sessions. Seventeen studies (57%) provided training to their chosen instructor before intervention. For the 17 teacher‐led interventions, 11 studies (65%) provided training for teachers prior to initiation of the intervention.

**TABLE 2:** Intervention characteristics (presented separately for studies in children and adolescents).

First author (publication date), study name	Theoretical framework	Setting and provider	Delivery and duration	Process evaluation	Primary outcome	Post/follow‐up intervention effect	Effects reported by Progress‐Plus factors on primary outcome
Children							
Ahmad [46] (2018), REDUCE	SCT	University Researchers	Face‐to‐face, Facebook, 4 months	No	BMI	No significant differences post intervention (*p* = 0.249) Significant differences between experiment group and wait‐list groups six‐month post training (*p* = 0.001, *p* < 0.001) respectively	Not reported
Bergh [47] (2012), HEIA	SEM, SCT	School Teachers	Face‐to‐face, 20 months	Yes	Psychological and social‐environmental determinants	A small reduction in self‐efficacy in the intervention group post intervention (*p* = 0.02)	No mid‐way interaction effects for gender or parental education on four determinants and no interaction effect of gender post‐intervention
Bernal [48] (2021), School Based	SEM	School Teachers	Face‐to‐face, 3 academic years	No	PA and SB	Increase in percentage of active children and low SB (p = 0.00) but no change during intervention and at 1‐year follow‐up	Not reported
Carson [49] (2013), Transform‐US!	SCT, BCT, SEM	School Teachers	Face‐to‐face, 18 months	Yes	SB	Significant mid‐intervention effect on total weekday sedentary time (*p* < 0.05)	Not reported
De Lepeleere [50] (2017), Movie Models	SDT, SCT	Online Researchers	Online, 4 weeks	Yes	PA, screen time, and healthy diet	No significant differences at 1‐month follow‐up (*p* = 0.099) and 4‐months (*p* = 0.59) follow‐up	Not reported
Faghy [51] (2021), Multi‐Component Education and Activity	COM‐B Model	School Teachers and researchers	Face‐to‐face, 12 weeks	Yes	PA	No significant differences at post (*p* > 0.05)	No differences by gender (p > 0.05)
Harrison [52] (2006), Switch‐Off‐Get Active	SCT	School Teachers	Face‐to‐face, 16 weeks	No	PA, screen time, and BMI	MVPA were higher in the intervention group (*p* < 0.05). Screen time was not significantly different (*p* = 0.13)	Not reported
Jago [53] (2013), Teamplay	SDT	Community center Researchers	Face‐to‐face, 8 weeks	No	Weekend MVPA	11 more minutes of weekend MVPA than the control group Effects not maintained at follow‐up	Not reported
Kipping [54] (2014), Active for Life Year 5	Not reported	School Teachers	Face‐to‐face, 6–7 months	No	MVPA, SB, and fruit and vegetable	No significance between experimental and control groups (*p* > 0.05)	Not reported
Moore [55] (2019), Healthy Change and System Change	Not reported	School Interventionists	Face‐to‐face and phone, 3 years	No	BMI	No significant differences in BMI over the 3‐year study (*p* > 0.05)	No differences by gender, race, or household income
Puder [56] (2011), Ballabeina	Not reported	School Teachers and health promoters	Face‐to‐face, 1 school year	Yes	BMI and aerobic fitness	Significant increase in aerobic fitness (*p* = 0.01) but no difference in BMI (*p* = 0.31)	Children with low educational level parents benefited less but not significantly (*p* > 0.05)
Robinson [57] (2021), Stanford GOALS	SCT	School Researchers	Face‐to‐face, 3 years	Yes	BMI	No significant differences in BMI over the 3‐year study (*p* > 0.45)	Not reported
Salmon [58] (2008), Switch‐Play/Switch‐2‐Activity	SCT, BCT, SEM	School Teachers	Face‐to‐face, 9 months	Yes	BMI	Significant decrease in BMI (*p* < 0.01) and maintained at 6‐ and 12‐month follow‐up (p < 0.05)	No differences by gender
Siegrist [59] (2018), JuvenTUM 3	SCT	School Teachers	Face‐to‐face, 18 months	No	PA	Significant increase in PA (*p* < 0.038)	Increase in PA for females (*p* = 0.054) and males (*p* = 0.033)
Tapia‐Serrano [60] (2022), School Based	SDT	School Teachers and researchers	Face‐to‐face, 2.5 months	No	Movement behaviors	No significant interaction effects (p > 0.05)	Not reported
Vilardell‐Davila [61] (2023), Eat and Activate Yourself Healthily	Not reported	School Nutritionists and physical educator	Face‐to‐face, website, and text messaging, 9 months	No	MVPA and screen time	No significant differences within or between groups for MVPA at 6‐ and 12‐month follow‐up. Significant difference on screen time decreases between groups at 12 months (*p* = 0.003)	Not reported
Adolescents							
Ahmed [62] (2022), Multi‐component school‐based	Not reported	School Researchers	Face‐to‐face, 12 weeks	No	PA and screen time	Significant increase in PA at 8 and 12 weeks in experimental group (*p* < 0.001) and average screen time was reduced from baseline to 8 and 12 weeks	Not reported
Aittasalo [63] (2019), Kids Out	SEM	School Teachers	Face‐to‐face, 3 weeks	Yes	PA	Increase in self‐reported data but no significance for accelerometer data at 4‐week follow‐up (*p* > 0.05)	Not reported
Bandeira [64] (2020), Fortaleca sua Saude	Not reported	School Teachers	Face‐to‐face, 4 months	No	Screen time	No significant differences post intervention (*p* > 0.05)	No significant differences by gender or age groups (*p* > 0.05)
Bonnesen [65] (2021), Health High Schools	SEM, SCT	School Teachers	Face‐to‐face, 9 months	Yes	Wellbeing	No significance (*p* > 0.05)	No sociodemographic differences
Champion [66] (2023), Health4Life	Not reported	School Teachers	Face‐to‐face, online, and application, 6 weeks	No	Big 6 risk factors	No between‐group differences at 24 months	Not reported
Choi [67] (2018), PASB	Not reported	University Nurse and therapist	Face‐to‐face, 9 weeks	No	PA and SB	Significant increase in PA (*p* = 0.043) and decrease in SB (*p* = 0.008) post intervention	Not reported
Filion [68] (2015), Stop my Smoking USA	Not reported	Text messaging Researchers	Text messaging, 6 weeks	No	Sleep quantity and quality	No significant differences at 3‐month follow‐up (*p* > 0.05)	Not reported
Knebel [69] (2020), Movimente	SEM, SCT	School Teachers and researchers	Face‐to‐face, 1 year	Yes	Total sleep time	No significance on weekdays (*p* = 0.827) and weekends (*p* = 0.163)	Not reported
Laska [70] (2016), CHOICES	SCT, SNT	College, online, and hybrid Researchers	Face‐to‐face, online, and hybrid, 24 months	Yes	Health behaviors	Significant reduction of fast food (*p* = 0.0007) but limited changes of other behaviors at 4‐ month follow‐up. Few differences observed at 12‐ and 24‐ month follow‐up	Marginal significant differences in ethnicity (*p* = 0.07)
Mauriello [71] (2010), Health in Motion	TTM	School Researchers	Online, 14 months	No	PA, fruit and vegetable servings, and TV viewing	Experiment group reported greater number of days participating in 60 min of PA at 2 months and ate significantly more fruit and vegetables consumption at 2, 6, and 12 months, but no differences on TV viewing	Not reported
Sevil [72] (2019), Paths of the Pyrenees	SEM, SDT, TPB	School Teachers	Face‐to‐face, 1 academic year	No	Multiple health behaviors	Significant improvement across multiple health behaviors (*p* > 0.05)	Larger effect size in males than females
Smith [73] (2014), ATLAS	SDT, SCT	School Teachers and researchers	Face‐to‐face, 20 weeks	Yes	Body composition	No significant differences for BMI, waist circumference, and body fat percentage	Not reported
Werch [74] (2011), Project Active	Not reported	School Personal fitness coaches	Face‐to‐face, 30 days	No	Primary health risks	3‐month post‐intervention showed significant decrease in alcohol consumption (*p* = 0.01) and increase in fruit and vegetables servings (*p* = 0.01)	Not reported
Young [75] (2022), PCOS Kind Mind Programme	Not reported	Medicine practice and online Interventionists	Face‐to‐face and online, 5 weeks	No	Psychological distress, mindfulness, PA strategies, nutrition, and PA self‐efficacy	Significant improvement in higher nutrition self‐efficacy (*p* = 0.013), PA self‐efficacy (*p* = 0.0028), and PA strategies (*p* = 0.04) at intervention end	Not reported

Abbreviations: BlCT, behavioral choice theory; COM‐B, capability, opportunity, and motivation behavior; MVPA, moderate‐vigorous physical activity; PA, physical activity; SB, sedentary behavior; SCT, social cognitive theory; SDT, self‐determination theory; SEM, socioecological model; SNT, social network theory; TPB, theory of planned behavior; TTM, transtheoretical model of behavior change.

Intervention duration ranged from 3 weeks to 3 years; median duration was 18 months. All studies assessed outcomes at intervention end; a further 12 (40%) conducted follow‐up assessments, of which nine (30%) had multiple follow‐up assessments. Post‐intervention follow‐up duration ranged from 4 weeks to 3 years; median duration was 18 months. Eleven studies (37%) did not report using a theoretical framework to inform intervention design, 10 (33%) used one theoretical framework, and nine studies (30%) used multiple theoretical frameworks. Social cognitive theory was the most commonly used theoretical model (*n* = 12, 40%).

#### 3.2.2 Targeted Behaviors and Behavior Change Techniques

**FIGURE 2:**
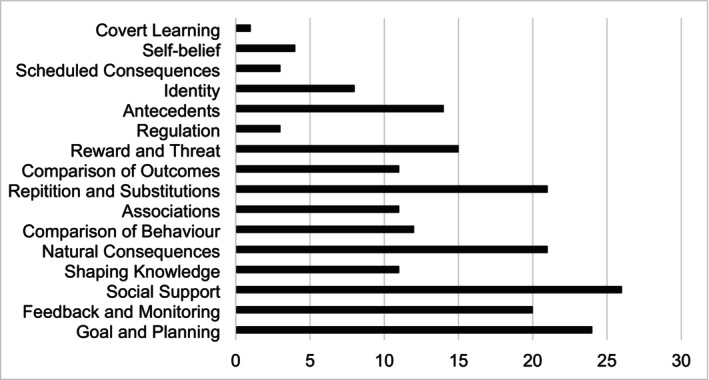
Frequency of use for the 16 clustered behavior change techniques [45].

Targeted behaviors are presented in Table 3. All 30 studies targeted physical activity, 25 (83%) targeted sedentary behavior, and 13 (43%) targeted sleep. The most common combination of target behaviors was physical activity and sedentary behavior (*n* = 9, 30%). The 93 behavioral change techniques were classified into 16 hierarchical cluster groups; the frequency by which each technique was used is summarized in Figure 2. Of the 16 hierarchical clusters, most studies used social support (*n* = 26, 87%), goals and planning (*n* = 24, 80%), natural consequences (*n* = 21, 70%), repetition and substitutions (*n* = 21, 70%), and feedback and monitoring (*n* = 20, 67%). From the 93 distinct behavioral change techniques, 57 were coded once or more; the number of behavioral change techniques employed per intervention ranged from one to 43, with a median of 22. The most frequently coded techniques were social support [unspecified] (*n* = 21, 70%), information about health consequences (*n* = 20, 67%), behavioral practice/rehearsal (*n* = 16, 53%), goal‐setting behavior (*n* = 16, 53%), and action planning (*n* = 14, 47%).

**TABLE 3:** Combinations of targeted behaviors.

PA	SB	Sleep	Diet	Smoking	Alcohol	Drug use	Reference
x	x						48, 49, 52, 53, 58, 63, 64, 69, 71
x	x		x				46, 47, 54, 57, 59, 61, 62, 73
x	x	x	x				51, 56, 60, 67, 70
x		x	x				65
x		x					50, 68, 75
x	x	x					55
x	x	x	x	x	x		66, 72
x		x	x	x		x	74

Abbreviations: PA, physical activity; SB, sedentary behavior.

#### 3.2.3 Outcomes

The primary outcomes included changes in multiple health behaviors, such as physical activity and sedentary behavior (*n* = 13, 43%), body mass index (*n* = 6, 20%), physical activity (*n* = 4, 13%), sedentary behavior (*n* = 2, 7%), sleep (*n* = 2, 7%), well‐being (*n* = 1, 3%), psychological distress and self‐efficacy (*n* = 1, 3%), and psychological and social‐environmental determinants (*n* = 1, 3%). Sixteen studies (53%) reported a significant effect on their primary outcome post‐intervention, one intervention reported significant effects at mid‐intervention, and four studies (13%) reported a significant effect at follow‐up. Twelve studies (40%) conducted a process evaluation.

### 3.3 Equity‐Denied Populations

Most studies (*n* = 20, 67%) did not test for differential effects by population subgroup. Of the 10 studies (33%) that explored differential effects, seven studies examined differences by sex and four each by ethnicity and socioeconomic position. No between‐group differences were observed in all cases. Six (20%) interventions were targeted at a specific “at‐risk” population, such as participants of low socioeconomic position or an ethnic minority group. From the 17 protocols available, six stated they would report differential effect sub‐group analysis, but only three of these studies did.

## 4 Discussion

This scoping review summarizes characteristics of combined movement behavior interventions in children and adolescents and describes recruitment and effectiveness patterns in equity‐denied population groups. The majority of studies have been conducted in Europe, completed within the last 5 years, and focused on children aged 5–11 years. Most studies targeted two movement behaviors in combination, mainly physical activity and sedentary behavior; few collectively targeted physical activity, sedentary behavior, and sleep. Interventions were predominantly delivered face‐to‐face by teachers in schools. Many studies described participant characteristics by PROGRESS‐Plus factors, but fewer than half examined whether intervention effects differed across these groups.

To date, most interventions in this field have been delivered in the school setting with teachers as the provider. Schools offer access to equity‐denied populations, such as low‐income families, and ethnic or religious minority groups [76], and are widely seen as an optimal environment to facilitate behavior change, with teachers well‐positioned to deliver intervention content [77, 78, 79]. However, teachers often report that pre‐service training does not provide them with the required skills or knowledge to implement health promotion interventions [80, 81]. As such, pre‐intervention training remains integral to the success of school‐based health promotion interventions [82], but just 11 of the 17 teacher‐led interventions in this review provided training for teachers prior to intervention delivery. Therefore, if schools and teachers are to remain the primary setting and provider for combined movement behavior interventions, future research may need to consider how to support and train teachers to promote behavior change.

Our review identified that few interventions to date have targeted all three movement behavior domains. While physical activity was targeted in every study, and most targeted sedentary behavior (83%), only 43% of studies targeted sleep. The limited focus on sleep identified here is consistent with previous reviews of school‐based movement behavior interventions [27]. Chung et al. [83] contend that schools are an ideal setting for the delivery of sleep behavior change programs as they can disseminate evidence‐based programs to children via the school curriculum. However, previous reviews have indicated that school‐based sleep education interventions have typically relied on sleep experts to deliver sleep content [84, 85], potentially adding cost and resource burden that may hinder large‐scale implementation. This reliance on specialized expertise potentially complicates the integration of sleep behavior into combined movement behavior interventions. Moreover, while physical activity and sedentary behavior change can be integrated into the school day, via active breaks for example, interventions typically aim to enhance sleep through education [85], without a comparable practical component. Future research may need to explore innovative strategies to address these challenges, which potentially include providing sleep‐related training for teachers or supporting parents to establish optimal sleep routines at home.

Of the 30 included studies, just 10 (33%) examined whether intervention effectiveness differed across participant sub‐groups, despite all included studies collecting at least two participant characteristics (e.g., age and gender) at baseline. From the 10 studies, no significant differences were observed across PROGRESS‐Plus subgroups. The limited exploration of differential effects is consistent with previous reviews of physical activity interventions [86, 87]. It is worthwhile to acknowledge that a small number of included studies (*n* = 6, 20%) targeted their intervention at a specific “at‐risk” population, such as participants of low socioeconomic position or an ethnic minority group. While such studies actively seek to reduce inequalities in the outcome of interest, there may be scope to examine differential effectiveness between subgroups that were not the primary focus, serving to ensure that even within such targeted interventions, some participants do not disproportionally benefit more or less than other population groups. A challenge to obtaining better evidence on the differential effectiveness of behavior change interventions is the well‐known limitation of subgroup analysis, which includes issues of statistical power, multiple hypothesis testing, and “data dredging” [88]. These issues have been addressed in recent guidance pertaining to subgroup analyses by Breck and Wakar [89]. Recommendations included, but were not limited to, appropriately specifying subgroup analyses as exploratory or confirmatory in nature, defining subgroups and related comparisons during the study phase and, relatedly, calculating minimum necessary sample sizes for such comparisons during the design phase. Subject to data availability, differential effectiveness can also be addressed via individual participant data meta‐analyses, such as that reported by Hartwig et al. [90]

In the studies included in this review, self‐directed behavior change techniques were the most commonly used, such as providing information about health consequences, instructions to perform, and setting goals, rather than adapting the physical or social environment. This is consistent with previous interventions targeting childhood obesity [91] and dietary intake [92]. Prior research indicates that “high‐agency” behavior change techniques, such as providing information, guidance, or instruction, may be insufficient to bring about behavior change if delivered in isolation [28]. Moreover, there are equity concerns of high‐agency interventions [93, 94]. For example, high‐agency techniques may be easier to understand for young people with better health literacy who often tend to have more affluent backgrounds [28]. A valuable next step for research on this topic would be to develop and evaluate low agency interventions, such as modifying the built environment, as these have been insufficiently tested [28].

The present review highlights the restricted range and application of combined movement behavior interventions outside the realm of promoting physical health or behavioral outcomes. Observational evidence indicates that movement behaviors may be beneficially associated with academic achievement [11, 95], and reductions in stress [96], anxiety and depressive symptoms [97], for example, but the impact of combined movement behavior interventions on these outcomes has not been tested widely to date. One potential avenue for future research would be to explore the impact of movement behavior interventions on mental health, academic achievement, or climate‐related outcomes, such as carbon emissions, for example. Evidence indicates that promoting active transportation, including walking or cycling, could reduce carbon emissions from transport by 67% [98]. Beyond the environmental advantages, the opportunity to concurrently enhance physical activity and reduce sedentary behavior could foster both environmental sustainability and individual health [99].

We acknowledge the following strengths and limitations of this scoping review. Our protocol was registered with Open Science Framework and reported in accordance with Preferred Reporting Items for Systematic Review and Meta‐Analysis extension for Scoping Reviews [33]. We used broad search criteria to identify relevant articles across electronic databases, supplemented with manual searches. The inclusion of gray literature, consistent with best practice guidelines, enhances the comprehensiveness of this review by capturing a wider spectrum of evidence. The established PROGRESS‐Plus framework [44] and Behaviour Change Technique Taxonomy Version 1 [45] were used to assess differential effectiveness and behavior change components used within the included studies. A limitation of this study was the restriction to English‐language only publications, but previous research indicates that this is unlikely to introduce bias in the included studies [100]. In some instances, coding of behavior change techniques was hindered by limited reporting of intervention characteristics, potentially resulting in the incorrect classification of behavior change techniques used or some intervention components being omitted.

### 4.1 Future Research

Key recommendations for future combined movement behavior interventions include:

- Develop strategies to support and train teachers in delivery of behavior change interventions.
- Develop and evaluate combined movement behavior interventions that explicitly assess differential effectiveness to ascertain whether they may lead to the widening of inequalities.
- Develop and test the effect of combined movement behavior interventions on outcomes other than physical health or behavioral domains, such as mental health, academic achievement, and climate‐related outcomes.
- Develop and test low‐agency combined movement behavior interventions.

## 5 Conclusion

This scoping review summarizes the characteristics of combined movement behavior interventions for young people. Our findings show that most interventions were implemented to improve behavioral or physical health‐related markers, with limited attention to outcomes outside of these domains, such as mental health or academic achievement. The extent to which movement behavior interventions may impact social or demographic inequalities across the spectrum of outcomes remains unknown and should be a priority for future research in this area.

## Conflicts of Interest

The authors declare no conflicts of interest.

## Supporting information


**Table S1** Preferred Reporting Items for Systematic reviews and Meta‐Analyses extension for Scoping Reviews (PRISMA‐ScR) checklist.
Table S2 Example search strategy.
